# Follicular dendritic cell sarcoma of the uterine cervix: a case report

**DOI:** 10.1186/s12905-020-01045-y

**Published:** 2020-08-17

**Authors:** Takuto Nakamura, Masato Yoshihara, Satoshi Tamauchi, Hiroaki Kajiyama, Fumitaka Kikkawa

**Affiliations:** 1 Department of Obstetrics and Gynecology, Anjo Kosei Hospital, Anjo, Aichi Japan; 2 Department of Obstetrics and Gynecology, Nagoya University Graduate School of Medicine, 65, Tsuruma-cho, Showa-ku, Nagoya, Aichi Japan

**Keywords:** Follicular dendritic cell sarcoma, Cervical cancer

## Abstract

**Background:**

Follicular dendritic cell sarcoma (FDCS) is a rare mesenchymal tumor that mostly occurs in systemic lymph nodes. FDCS in the uterine cervix has not yet been reported.

**Case presentation:**

A 49-year-old woman was referred to our department with a cervical tumor, which was histologically suspected to be undifferentiated carcinoma. She underwent hysterectomy, salpingo-oophorectomy, and pelvic lymphadenectomy after neoadjuvant chemotherapy with paclitaxel and carboplatin. The resected specimen contained high numbers of spindle cells and was immunohistochemically confirmed to be FDCS. The tumor was completely resected and recurrence was not detected at a 16-month follow-up.

**Conclusion:**

FDCS is an extremely rare malignant tumor in the uterine cervix, and an accurate diagnosis and complete resection are essential for a good prognosis.

## Background

Follicular dendritic cell sarcoma (FDCS) is a rare mesenchymal tumor that was initially reported in 1986 [1]. Three types of tumors are derived from dendritic cells: FDCS derived from follicular dendritic cells that present antigens to B lymphocytes in lymph follicles [2], interdigitating dendritic cell sarcoma derived from the T-cell zones of lymphoid organs, such as the paracortex and deep cortex of the lymph nodes, and fibroblastic reticular cell sarcoma derived from a reticular network of lymphoid organs [3]. An accurate diagnosis is challenging without an appropriate series of immunohistochemistry; therefore, some tumors may be diagnosed as undifferentiated carcinoma. The accuracy of a diagnosis influences the prognosis of patients because of the low response rate to established chemotherapy and radiotherapy [3–5].

Although previous studies described FDCS in the cervical lymph nodes, liver, stomach, and tonsils, FDCS in the uterine cervix has not yet been reported. We herein present a 49-year-old woman diagnosed with FDCS in the uterine cervix, which was successfully treated by complete surgery without postoperative adjuvant therapy based on a precise pathological diagnosis. This is the first case of FDCS arising from the uterine cervix, and we described the time course of this patient from her initial admission to diagnosis and treatment. We also emphasized the importance of considering FDCS as a differential diagnosis for cervical tumors because an accurate diagnosis and complete resection are essential for a good prognosis.

## Case presentation

**Fig. 1:**
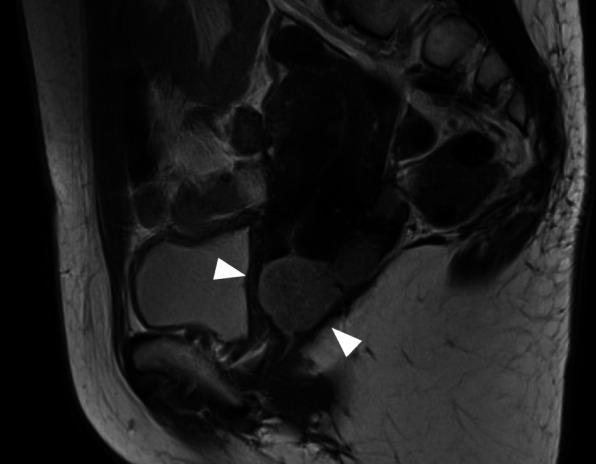
Magnetic resonance imaging of the patient at the initial presentation. A solid tumor developing from the posterior of the uterine cervix was confirmed (arrowheads)

A 49-year-old woman, gravida 4 para 2, with abnormal genital bleeding was referred to our department with a cervical mass. She had a previous history of uterine myomectomy and cervical polypectomy, which were both benign diseases. Pelvic magnetic resonance imaging revealed a 28 × 25 mm solid tumor developing from the posterior of the uterine cervix (Fig. 1), and 28 × 24 and 21 × 24 mm space-occupying lesions were also detected around the rectus, which were suspected to be lymph node metastases. Chest-abdominal computed tomography (CT) showed no enlarged lymph nodes or distant metastasis, except for the tumors already detected. Positron emission tomography-CT revealed the accumulation of fluorodeoxyglucose (maximum standardized uptake value, 6.60) at the cervical tumor only. Serum tumor marker levels were as follows: carcinoembryonic antigen, 2.0 ng/ml; cancer antigen-125, 158.8 U/mL;cancer antigen-19-9, 137.2 U/ml; squamous cell carcinoma antigen, 0.5 ng/ml; soluble interleukin-2 receptor, 199.0 U/ml. The other results of the blood examination, including renal function, liver enzymes, and electrolytes, were within normal limits.

Biopsy of the cervical tumor was performed and a histopathological examination indicated that the tumor had atypically spindle, ovoid, and polygonal cells with an eosinophilic hyaline-rich cytoplasm, with whorled and cord-like forms. Nuclei were oval to round and had mild atypia. Inflammatory cells were scattered to various degrees around tumor cells. The results of immunohistochemical staining were as follows: cytokeratin AE1/AE3, positive; epithelial membrane antigen (EMA), negative; CAM5.2, negative; cytokeratin-MNF116, positive; S-100, negative; vimentin, positive; actin, negative; desmin, negative; cluster of differentiation (CD) 3, negative; CD8, negative; CD10, negative; CD5, negative; CD20, negative; CD79a, negative; PgR (+), PgR (+), inhibin, negative; thyroid transcription factor-1, negative; Epstein-Barr virus-encoded RNA in situ hybridization, negative.

**Fig. 2:**
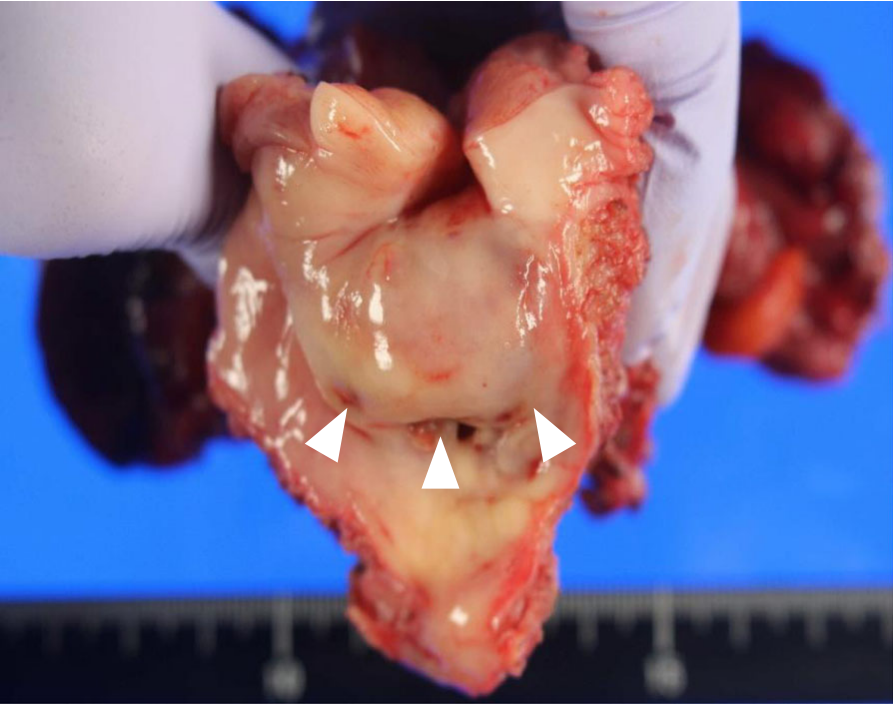
Surgical specimen of the tumor. The tumor with a necrotic lesion arose from the posterior side of the cervix (arrowheads)

**Fig. 3:**
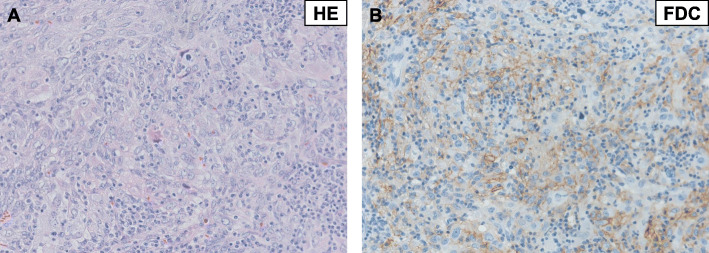
The histopathology of the cervical tumor. The tumor had atypically spindle, ovoid, and polygonal cells with an eosinophilic hyaline-rich cytoplasm, with whorled and cord-like forms. Nuclei were oval to round and had mild atypia. **a**. Tumor cells were positive for FDC (**b**)

Since the tumor was suspected to be undifferentiated carcinoma of the uterine cervix, the patient received chemotherapy with four cycles of paclitaxel (180 mg/m^2^) and carboplatin (AUC5, 700 mg/body). Massive genital bleeding occurred 5 months after the admission; therefore, the patient underwent extended total hysterectomy, bilateral salpingo-oophorectomy, and pelvic lymphadenectomy. Intraoperative findings revealed a small amount of bloody ascites and the resected specimen had a necrotic mass at the posterior side of the cervix (Fig. 2). Tumor cells exhibited the same histopathological characteristics as those in the previous biopsy (Fig. 3a). Additional immunohistochemistry revealed cells that were positive for both CD68 and FDC (Fig. 3b), which was consistent with the diagnosis of FDCS. The tumor was completely resected and lymphovascular invasion was not detected. There was also no evidence of lymph node metastasis. She did not receive adjuvant therapy and her CA19–9 level decreased to within almost normal limits (Table 1). Tumor recurrence was not detected at the 26-month follow-up after admission. The consent of the patient for publication was recorded according to the Ethics Committee of Nagoya University and the principles of the Declaration of Helsinki.

**Table 1:** Level of tumor markers in each follow-up period

Tumor marker (Normal range)	Admission	At surgery (6 m after admission)	At final follow-up (26 m after admission)
**CA-125, U/mL** (< 35.0)	158.8	40.8	11.5
**CA-19-9, U/mL** (< 37.0)	137.2	53.0	40.0
**CEA, U/mL** (< 5.0)	2.0		
**SCC, ng/mL** (< 1.5)	0.5		
**sIL-2R, U/mL** (121.0 ~ 613.0)	199.0		

Abbreviation: *CA* cancer antigen, *CEA* carcinoembryonic antigen, *SCC* squamous cell carcinoma antigen, *sIL-2R* soluble interleukin-2 receptor

## Discussion

This is the first case of FDCS in the uterine cervix. After reported surgery and chemotherapy, we reached a final diagnosis. Our experience demonstrates the difficulties associated with accurately diagnosing FDCS due to a lack of familiarity with the pathologies of rare tumors, including sarcoma. Although FDCS in the gynecological system is markedly rarer than other carcinomas, it needs to be considered as a differential diagnosis because of its potentially fatal prognosis without appropriate treatment.

FDCS has been reported in approximately 500 cases worldwide with an age range of 10 to 90 years and a male:female ratio of 1:1. The primary lesion of FDCS is mostly in the lymph nodes in the neck, axilla, and mediastinum. The remainder of FDCS originate from extra-lymphatic lesions, such as the liver, mesenterium, stomach, small intestine, pharynx, tonsils, retroperitoneum, and ovary [3–5]; however, FDCS in the uterine cervix has not yet been reported in the English literature.

Due to the wide range of primary lesions, there is no known specific initial symptom of FDCS; some patients may exhibit symptoms associated with an increase in tumor volume, such as lymph node swelling [6]. Since there are no specific findings in blood examinations or imaging studies, difficulties are associated with making an accurate diagnosis prior to the initiation of treatment. The diagnosis of FDCS is pathologically confirmed using biopsy or resected specimens; however, due to the rarity of this tumor, it is important to conduct appropriate immunohistochemistry in consideration of FDCS from the findings of hematoxylin and eosin staining. The tumor cells of FDCS are spindle to epithelioid in shape with the coexistence of multinucleated cells. Single nuclear inflammatory cells infiltrate and form bundles, flowers and swirls structures. Some giant cells and Reed-Sternberg cells are also detected in these tumors. To reach a definitive diagnosis of FDCS, FDC markers, such as CD21, CD35, Ki-M4p, and CNA.42, are used to distinguish it from other tumors that may have a mesenchymal structure, such as undifferentiated carcinoma, meningioma, and paraganglioma [7, 8]. Alternatively, FDCS may be found within lesions of Castleman’s disease, particularly its hyaline-vascular type [5]. It has been reported that FDCS occurred after the excision of a lesion of hyaline-vascular type Castleman’s disease, and occupied most of the lesions. It is also important to distinguish FDCS from inflammatory pseudotumor-like FDCS associated with Epstein-Barr virus. This tumor is often found in the abdominal organs, particularly in the liver and spleen, and has the characteristics of positive FDCS markers and the detection of EBV by in situ hybridization. It progresses more slowly than FDCS, and long-term survival has been reported even after recurrence [9].

The treatment for cervical cancer principally involves surgery, radiation, and chemotherapy. New surgical techniques, such as laparoscopic radical hysterectomy and sentinel lymph node biopsy, have also become available [10, 11]. On the other hand, a basic therapeutic strategy for FDCS is prioritized to guarantee complete surgical tumor resection because of the low response rate to established chemotherapy and radiotherapy [3–6]. Saygin et al. examined 216 patients with FDCS and reported 2-year survival rates of 82.4, 80 and 42.8% for early, locally advanced, and distant metastasis disease, respectively. Patients who underwent complete tumor resection had a better prognosis than those with unresectable localized tumors. Furthermore, no prognostic difference was observed between the surgery group and postoperative radiotherapy groups. A large tumor size (larger than 6 cm) and lymphoplasmacytic cell invasion have been identified as poor prognostic factors [3]. In the present case, as the tumor remained localized and was completely resected, the patient could follow favorable clinical course without any tumor recurrence.

## Conclusion

FDC sarcoma is a rare tumor that may develop in the uterine cervix. FDCS needs to be considered when confirming a mesenchymal cervical tumor and appropriate immunohistochemistry needs to be performed for both an accurate diagnosis and selection of a therapeutic strategy.

## Data Availability

Not applicable.

## Abbreviations

FDC: Follicular dendritic cell sarcoma
FDCS: Follicular dendritic cell
CT: Computed tomography
CD: Cluster of differentiation
